# No xylem phenotypic plasticity in mature *Picea abies* and *Fagus sylvatica* trees after 5 years of throughfall precipitation exclusion

**DOI:** 10.1111/gcb.16232

**Published:** 2022-05-27

**Authors:** Giai Petit, Dario Zambonini, Benjamin D. Hesse, Karl‐Heinz Häberle

**Affiliations:** ^1^ Dipartimento Territorio e Sistemi Agro‐Forestali (TESAF) University of Padova Padova Italy; ^2^ Land Surface‐Atmosphere Interactions Technical University of Munich, School of Life Sciences Freising Germany; ^3^ Chair of Restoration Ecology Technical University of Munich, School of Life Sciences Freising Germany

**Keywords:** acclimation, drought, functional balance, phenotypic plasticity, phloem, precipitation exclusion, tree mortality, widening, xylem

## Abstract

Forest trees are experiencing increasing frequency and intensity of drought events with climate change. We investigated xylem and phloem traits from mature *Fagus sylvatica* and *Picea abies* trees after 5 years of complete exclusion of throughfall precipitation during the growing season. Xylem and phloem anatomy, leaf and branch biomass were analysed along top branches of ~1.5 m lenght in 5 throughfall precipitation excluded (TE) and 5 control (CO) trees of both beech and spruce. Xylem traits were analysed on wood cores extracted from the stem at breast height. In the top branches of both species, the lumen diameter (or area) of xylem and phloem conduits did not differ between TE and CO trees. At breast height, TE trees of both species produced narrower xylem rings and conduits. While allocation to branch (*BM*) and needle biomass (*LM*) did not change between TE and CO in *P. abies*, TE *F. sylvatica* trees allocated proportionally more biomass to leaves (*LM*) than *BM* compared with CO. Despite artificial drought increased the mortality in the TE plots, our results revealed no changes in both xylem and phloem anatomies, undermining the hypothesis that successful acclimation to drought would primarily involve increased resistance against air embolism.

## Graphical abstract

Drought events increasing in severity and frequency are threatening the survival of forest trees globally. The xylem and phloem phenotypic plasticity is supposed to be a key strategy to cope with drought stress. We investigated xylem and phloem traits from mature beech and spruce trees after 5 years of throughfall precipitation exclusion during the growing season. We found that the axial scaling of vessel and tracheid diameter, and of tracheid wall thickness did not undergo significant changes after the drought exposure. These results undermined the hypothesis that successful acclimation to drought would primarily involve increased resistance against air embolism.

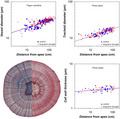

## 1 INTRODUCTION

In the last decades, the increasing frequency of extreme events, such as droughts and heatwaves, is occurring worldwide, and often has been recognized as triggers of phenomena of tree vigor decline and tree mortality in natural ecosystems (Allen et al., 2010, 2015; Schuldt et al., 2020). At present, the actual cascade of events affecting the tree physiological status preceding death under the effects of episodic droughts and chronic increase in temperature and atmospheric vapour pressure deficit has still to be thoroughly identified (McDowell et al., 2020). Most commonly, exceptional events of drought‐related tree mortality are perceived as sudden events. However, in most cases, they are preceded by long‐term signals, such as the long‐term reduction in diameter growth (Cailleret et al., 2017) and/or crown condition decline with dessication symptoms (Carnicer et al., 2011).

Trees are long living organisms that translocate aqueous solutions over very long distances through transport tissues of xylem and phloem, which are seasonally produced *ex novo*. Water flows along a gradient of negative water potential developing along soil–plant–atmosphere‐continuum (SPAC). Under high tension due to drought, air bubbles can seed through pit membranes and expand to fully embolize xylem conduits. This interrupts water transport and consequently decreases the total xylem hydraulic conductance (Cochard, 2006). The percentage loss of xylem conductance (*PLC*) for decreasing xylem water potential (*Ψ*_*xyl*_) (i.e., the so called vulnerability curves) depends on the different combination of anatomical traits of vascular elements (e.g., conduit length and diameter; pit size and density, etc.) (Hacke & Sperry, 2001). Therefore, the xylem vulnerability to drought‐induced embolism formation is species‐specific (Choat et al., 2012), but it has been also reported to vary across environmental settings (Barnard et al., 2011; Skelton et al., 2021).

In trees, drought conditions can negatively affect the maintenance of a positive C balance at the tissue or individual level by exerting limitations to the water transport sustaining leaf transpiration and photosynthesis. A theoretical framework has been proposed to explain the possible physiological failures causing tree death under drought (McDowell et al., 2008). More isohydric species maintain a safe margin between the *Ψ*_*xyl*_ at stomata closure and the critical *Ψ*_*xyl*_ at which air embolism would develop into xylem conduits (Choat et al., 2012; Meinzer et al., 2009). When isohydric species experience prolonged limitations to stomatal conductance, it is supposed that they can exhaust their internal carbon reserves, negatively affecting the overall C balance and possibly leading to death by carbon starvation (McDowell et al., 2008). On the other extreme, anisohydric species maintain stomatal conductance beyond the critical level for embolism formation, and *Ψ*_*xyl*_ can lower so much that the majority of its xylem conduits gets embolized. Consequently, the leaf water supply would be compromised by the hydraulic failure of the xylem transport system, with consequent crown desiccation and plant death (N. McDowell et al., 2008). The vulnerability to drought‐induced embolism formation often has been reported to be significantly related to the xylem conduit diameter (Hacke et al., 2006; Pittermann et al., 2006). However, this relationship is likely not causal but possibly arises from covariation with other functional traits, like pit size, number and density (Becker et al., 2003; Lazzarin et al., 2016). In the context of drought‐related tree mortality, the leaf water supply in trees exposed to lethal doses of drought has been commonly reported to be compromised by widespread air embolism in xylem conduits (Adams et al., 2017). Drought acclimation (i.e., the maintenance of a long‐term positive C balance) would imply that either the limitations to xylem and phloem transport are somehow compensated to maintain their hydraulic efficiency, or the C costs, get somehow reduced, especially those associated to the production of new biomass and the maintenance metabolism of the whole living tissues. Therefore, the assessments of species‐specific drought thresholds are currently recognized of key importance in order to better predict the effects of climate change on forest ecosystems (Brodribb et al., 2020).

Beyond the restriction in the water transport as a driver of drought‐induced tree vigor decline and mortality, also the limitations to phloem transport have been hypothesized to play a relevant role (Sevanto, 2014). The transport of sugars along the phloem is tightly coupled to the xylem water transport (De Schepper et al., 2013; Hölttä et al., 2006). Consequently, a drought‐induced reduction in xylem water potential should be accompanied by a contextual reduction in phloem water potential, which can be defined either passively by tissue dehydration or actively by tissue osmoregulation (i.e., by increasing the amount of osmolytes [primarily sugars and ions] in the phloem sap solution). In either case, phloem conductance would decrease due to the increase in sap viscosity (i.e., according to the Hagen Poiseuille law: Savage et al., 2017), possibly limiting the translocation of sugars from source to sink tissues (Sevanto, 2018). The C allocation to growth has been commonly known to be limited under drought conditions. Besides, a few recent studies reported higher leaf biomass in healthy trees from drier compared with moister conditions, suggesting that this modified biomass allocation can compensate for the negative effects of drought on gas exchanges and ultimately on the plant C balance (Anfodillo et al., 2016; Petit et al., 2016).

In this context, having a clearer panorama on the degree of phenotypic variability in those functional traits playing a key role in the survival of trees facing drought events is of primary importance. The concept of xylem and (to a minor extent) phloem phenotypic plasticity being key determinants of the acclimation potential of the different tree species is pervasive in plant ecology. Since the conductive system of both xylem and phloem is periodically renewed with the allocation of C into new xylem and phloem biomass, it is a common expectation that the newly formed xylem and phloem vascular elements differ in key anatomical features, such as lumen size, and thus provide better acclimation potentials to changing environmental conditions.

Recently, contradictory results have been found from anatomical studies applying different sampling approaches. Punctual anatomical analyses (fixed age or fixed diameter for sampling) typically revealed significant phenotypic trait variability in trees growing under different environmental conditions. Such studies mostly reported a more embolism resistant xylem with narrower conduits in trees from drier environments, as opposed to trees from moister environments showing larger and more conductive vascular elements with less resistance to embolism (Larter et al., 2017; Pfautsch et al., 2016; Schuldt et al., 2016). These types of evidence appeared to support the hypothesis of an existing trade‐off between embolism resistance and hydraulic conductivity (usually called safety vs. efficiency trade off), with resistance being prioritized under drier conditions and hydraulic efficiency supporting fast growth rate being prioritized under moister conditions (Sperry et al., 2008). Furthermore, anatomical analyses on tree cores typically showed that xylem cell production and conduit size are limited under drier environmental conditions (Castagneri et al., 2015, 2020). Most often analyses of functional xylem anatomy are punctual, that is they report data from samples extracted either at the stem base or from branches of fixed age (e.g., 2–3 years) or diameter (e.g., 1–2 cm). This type of analysis is neglecting the axial distance from the apex, which has been shown to be the primary driver of xylem anatomical variations (e.g., conduit diameter: Anfodillo et al., 2013; Olson et al., 2014; vessel clustering: Lechthaler et al., 2019; pit ultrastructures: Christof et al., 2020; Lazzarin et al., 2016; Pfautsch et al., 2018).

On the contrary, analyses removing or accounting for path length effects supported the hypothesis of a marginal degree of phenotypic plasticity, if any. In recent years, it has been documented that the assessment of phenotypic plasticity in anatomical traits is a delicate operation, because rather rigid axial designs characterize both xylem (Anfodillo et al., 2013; Lechthaler et al., 2019) and phloem (Kiorapostolou et al., 2020; Kiorapostolou & Petit, 2019) anatomies. Power scaling relationships

$Y=a\times X^{b}$

with exponent *b* ~ 0.1 to 0.3 have been commonly reported to well describe the axial variation in xylem and phloem conduits with the increasing distance from the apex at both the stem (Anfodillo et al., 2013) and branch level (Petit et al., 2016). Accordingly, the increase in conduit diameter is sharp within a few meters from the apex, but below it typically approaches a more constant size towards the stem base (Mencuccini et al., 2007; Petit et al., 2010). Without implying variations in the allometric constant *a*, conduit diameter would be larger either at the stem base of taller trees (Olson et al., 2014) and at given cambial age in fast growing trees (Carrer et al., 2015), simply because of the occurrence of the axial conduit diameter variation (*b*). Ecological studies applying such an allometric approach to anatomical analyses reported a completely different scenario, than punctual analysis/studies. When accounting for the effect of tree height, the xylem conduit diameter at the stem base was reported either not to vary across precipitation gradients (Fajardo et al., 2020) or even to increase with increasing VPD (Olson et al., 2020). Consistently, more detailed analyses reporting conduit diameter variations along the longitudinal axis of stem or branches showed invariant axial scaling exponents *b* (i.e., the rate of variation with increasing distance from the apex), but either invariant or higher allometric constant *a* (i.e., larger diameters all along the longitudinal stem/branch axis) under drier conditions (Guérin et al., 2018; Kiorapostolou & Petit, 2019; Lechthaler et al., 2019; Petit et al., 2016).

The context of this study is a throughfall exclusion experiment in the Kranzberg forest in south‐east Germany. Due to the throughfall exclusion system, a mature stand of intermixed *Picea abies* Karst. and *Fagus sylvatica* L. has been drought stressed for five consecutive growing seasons (2014–2018, Grams et al., 2021). This resulted in a strong decrease in physiological functionality for both species (Grams et al., 2021; Tomasella et al., 2018). Mortality events increased under drought by 7.5% and 1.5% in the coexisting spruce and beech trees, respectively (Pretzsch et al., 2020). The specific aim of this study was to apply the allometric approach to assess potential anatomical changes under drought in both species:

- By producing modified xylem and phloem structures to compensate for the hydraulic limitations in the long distance transport of water and sugars.
- By adjusting the allocation patterns of the new foliage and branch biomass to reduce the C costs associated with biomass production and maintenance.

Specifically, we assessed power scaling relationships (*Y = a × X* ^*b*^) of xylem, phloem and leaf/needle traits vs. distance from the branch apex. Trait conservativism would occur when both allometric parameters (*a* and *b*) do not differ between treatments, whereas plastic modifications would occur when *a* and/or *b* differ in drought stressed vs. control trees.

## 2 MATERIALS AND METHODS

### 2.1 Study site and plant material

The study site (Kranzberg Forest, Southern Bavaria, Germany; N48°25'12", E11°39'42"; elevation: 490 m a.s.l.) is a mixed stand of mature Norway spruce (*Picea abies* Karst.) and European beech (*Fagus sylvatica* L.) with an average height of ~30 m. Air temperature has an annual mean of 7.8°C and a growing season average of 13.8°C (May–September). The total annual precipitation is 750–800 mm, whereas during the growing seasons is 460–500 mm (Grams et al., 2021). In spring 2010, 12 plots of 110–220 m^2^ including 3–7 *P. abies* and 3–7 *F. sylvatica* trees were trenched along the perimeter down to 1 m (reaching a dense clay layer), and ditches were subsequently impermeabilized with plastic tarp impermeable to root growth and refilled with soil (Pretzsch et al., 2016).

Since May 2014, rainfall has been excluded from six plots by means of automated roofs at ~3 m aboveground, closing in case of precipitation during the growing season (i.e., approximatively from the mid of April until the mid of November) and reducing the annual throughfall by about 70% on the treatment plots. A detailed description of the experimental site and design can be found in Grams et al. (2021). Trees growing under drought experienced severe drought stress for five consecutive growing seasons with pre‐dawn water potentials as low as −1.8 MPa and soil water content close to the permanent wilting point (Grams et al., 2021). Target trees were identified in three roofed (Throughfall Exclusion, TE: drought stress treatment) and three unroofed (control: CO) plots, and their upper crown was accessible through a canopy crane.

### 2.2 Branch xylem and leaf biomass sampling

For both *P. abies* Karst. and *F. sylvatica* L. trees, a 1.5–2 m long branch was sampled from 5 TE and 5 CO trees in 2018. Sampling was designed to minimize the possible occurrence of anatomical adjustements related to crown position (Bettiati et al., 2012) and mechanical support (i.e., reaction wood). Along the main axis of each branch, 5–8 sampling points, numbered progressively starting from the most apical one, were selected at 1 cm above the base of each visible internode. The correct calendar year of formation was assigned to each internode and its length (*ΔL*) was measured. Instead, since annual increments in *F. sylvatica* were not precisely identifiable by visual inspection of internodes, for this species *ΔL* was estimated as the distance (*l*) between two sampling positions along the branch divided by the difference in the number of their yearrings in the xylem (*ΔL* = *l*/*ΔN*_*RINGS*_). For each point, the distal distance to the branch apex in previous years was then calculated by subtracting the corresponding *ΔL*s to *DA*. A segment of ~1.5 cm was cut at each sampling location and enclosed into a 50 ml container filled with a solution of 50% ethanol in distilled water for the following anatomical analyses. Starting from the first (i.e., most apical) sampling point, all the distal leaves/needles (*LMd*) were removed and placed in a labelled paper bag together with the total distal branch biomass (*BMd*). The sampling of the remaining *LMd* and *BMd*, and their placement into the respectively labelled paper bag proceeded progressively to the next sampling point towards the base. Paper bags containing *LMd* and *BMd* were then oven‐dried at 72°C for 24 h. The amount of leaves/needle (*LM*) and branch biomass (*BM*) for each sampling point was calculated as the cumulative sum of *LMd* and *BMd*, respectively, starting from the apical sampling point. Furthermore, for each branch, a subset of ~20 leaves/needles were placed in a separate paper bag and scanned before drying in the oven. Images were then analysed with ImageJ (Schindelin et al., 2012) and the total leaf area was measured. The ratio of leaf/needle dry mass: area *(LMA*) was then assessed for each subset of leaves/needles. For each sampling point of each branch, the total leaf area cumulated starting from the apex was then calculated as *LA = LM/LMA*.

### 2.3 Xylem core sampling at breast height

From the base of each sampled tree, a wood core to the pith was extracted from the stem at breast height with an increment borer (diameter = 0.5 cm). Together with the branch segments, the cores were sent to the laboratory of the Dept. LEAF of the University of Padua (Italy) for the following anatomical analyses.

### 2.4 Anatomical analyses on stem and branch xylem/phloem

Cores have been first cut into segments of max. 4 cm, which were then rehydrated for 10 min at high vapour pressure into a pressure cooker. Core segments were then mounted on a custom‐made clamping support designed for core transversal sectioning. Branch segments were removed from the preserving ethanol solution and directly mounted on the microtome clamping support. Each core and branch segment was cut at 15–20 μm thickness with a rotary microtome (Leica RM2245; Leica Biosystems, Nussloch, Germany), stained with a solution of Safranin Astra Blue (1% and 0.5% in distilled water, respectively) and permanently fixed on glass slides with Eukitt (BiOptica, Italy).

Images of the entire cross‐section of each core and branch segment were acquired with a Dsight slide scanner (Menarini Group, Florence, Italy) at 100× magnification. Image analysis was performed with ROXAS (von Arx & Carrer, 2014). The analysis was performed on a known angle *α* of 20–60°. The software required an outlining of the annual ring borders, used to assign the correct calendar year (*n*) and then automatically measured several anatomical traits at ring scale: those used in this study were the ring area (*RA*), the hydraulically weighted mean xylem conduit diameter (*DH = ∑d* ^*5*^ */∑d* ^*4*^, where *d* is the diameter of the *n*‐conduit, (Kolb & Sperry, 1999), the mean cell wall thickness of the conduits (*CWT*, μm) and the ring hydraulic conductivity (i.e., the sum of the conductivity of each conduit, calculated with Hagen–Poiseuille [Tyree & Ewers, 1991]: *KHr*). Data of *RA* and *KHr* were rescaled to the full cross‐sectional area by multiplying them by 360/*α*. Phloem mean cell area (*CA*_*PHL*_, μm^2^) was also obtained through ROXAS by measuring the largest 20–30 sieve elements in the non‐collapsed area of the phloem.

### 2.5 Statistical analyses

We tested for the differences between treatments and species in several allometric scaling relationships using linear mixed‐effects models fitted with restricted maximum likelihood (REML). Statistical analysis was made by using the lme4 package (Bates et al., 2015) of the software R (R Core Team, 2022). Data were first log_10_‐transformed to accomplish the assumption of normality and homoscedasticity (Zar, 1999). For each target trait, we tested for the fixed effects of the distance from the apex (*DA*) and treatment (TE/CO), and their interaction, using the tree ID as a random factor in all initial models. The best model was chosen based on Akaike Information Criterion (AIC) using the maximum likelihood method (Zuur et al., 2009).

## 3 RESULTS

### 3.1 Xylem and phloem anatomy in branches

**FIGURE 1:**
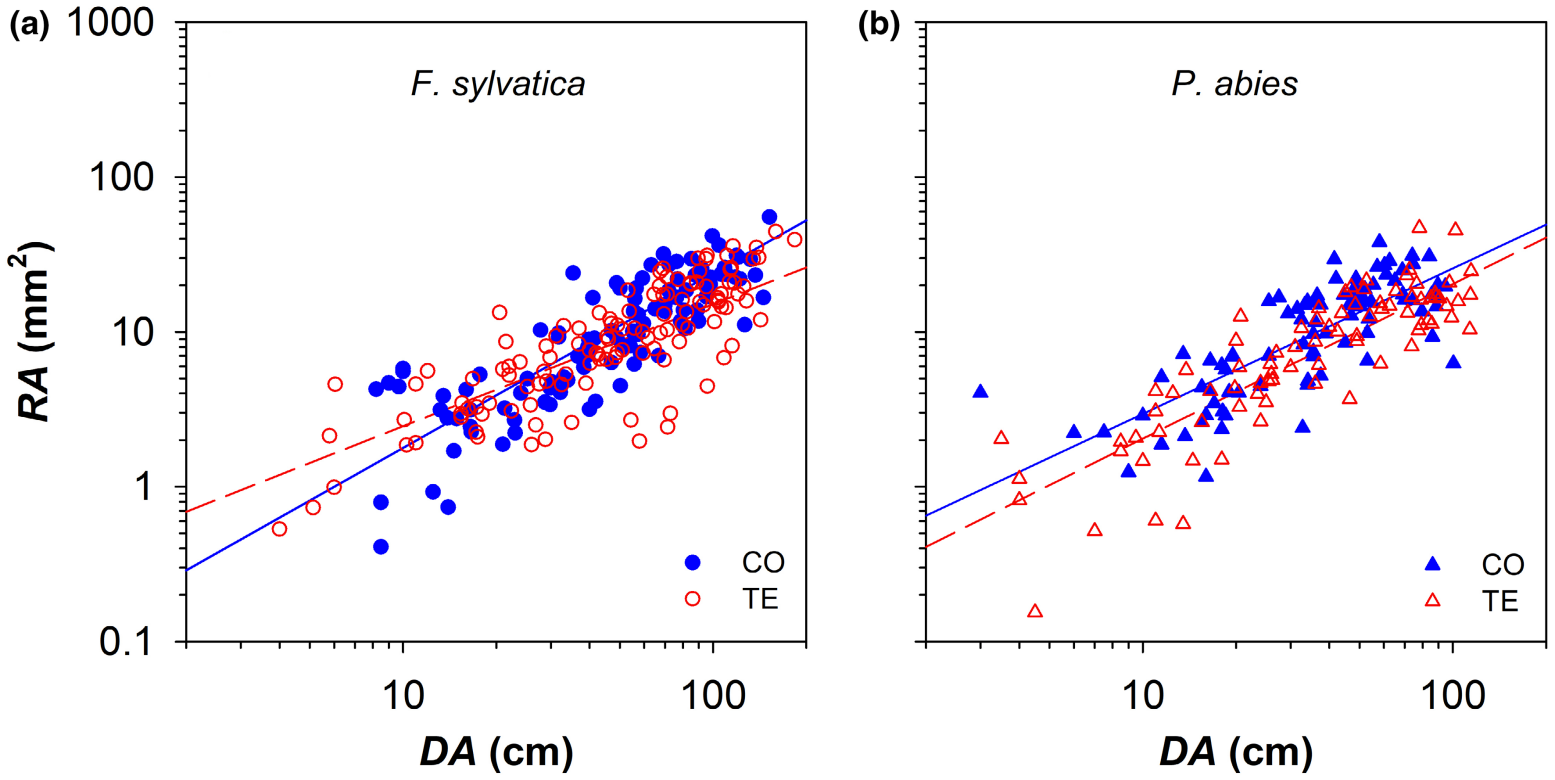
Variation in the ring area (*RA*) with the distance from the branch apex (*DA*) in control (CO: Filled blue symbols) and droughted (TE: Empty red symbols) for (a) *F. sylvatica* (circles) and (b) *P. abies* (triangles). Fitting lines (solid for CO and dashed for TE) are according to Table 1A.

**FIGURE 2:**
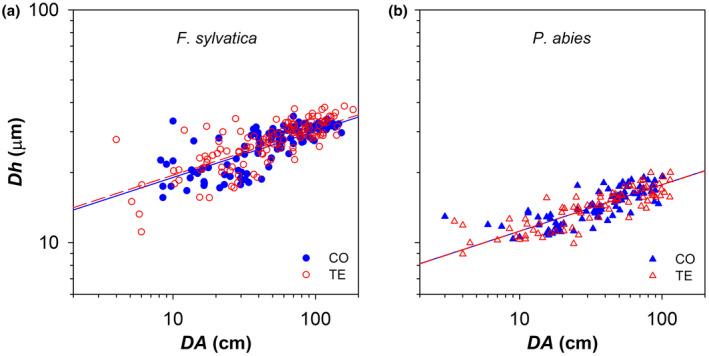
Variation in the mean hydraulically weighted diameter of xylem conduits (*dh*) with the distance from the branch apex (*DA*) in control (CO: Filled blue symbols) and droughted (TE: Empty red symbols) for (a) *F. sylvatica* (circles) and (b) *P. abies* (triangles). Fitting lines (solid for CO and dashed for TE) are according to Table 1B.

The five last annual xylem rings (from 2014 to 2018) showed clear axial patterns of ring area (*RA*, Figure 1) and hydraulically weighted conduit diameter (*Dh*, Figure 2). *RA* increased with the distance from the branch apex (*DA*) according to a nearly isometric scaling (*b* ~ 1) in both *F. sylvatica* and *P. abies*. The statistical models revealed significant variability for both the *y*‐intercept (corresponding to log_10_ *a* of power scaling equation, Equation 1: *Y = a × X* ^*b*^) and slope (corresponding to the exponent *b* of Equation 1) only for *F. sylvatica*, while *RA* resulted not significantly affected by precipitation exclusion in *P. abies* (Figure 1; Table 1A).

**TABLE 1:** Results of the optimal linear mixed‐effect models predicting the effects of log_10_ *DA* and *species + treatment* on (A) log_10_ *RA*, (B) log_10_ *Dh* and (C) log_10_ *CWT* (assessed for *P. abies only*). Tree ID was used as random factor in models, except (A) where nested random factors were ID/year. Abbreviations for species and treatments: FSCO Fagus Sylvatica COntrol, FSTE Fagus Sylvatica throughfall exclusion, PACO Picea Abies COntrol, PATE Picea Abies throughfall exclusion

Model	Fixed effects and covariates	Estimate	SE	*df*	*t*‐value	*p*‐value
(A) log_10_ *RA* ~ log_10_ *DA* + (species + Treat) random ID/year	Intercept (FSCO)	−0.88	0.10	292	−8.64	<.0001^A^
	log_10_ *DA* (slope) (FSCO)	1.13	0.05	292	21.41	<.0001^a^
	FSTE	−0.40	0.14	18	3.46	.0028^B^
	PACO	−0.47	0.15	18	2.73	.0136^B^
	PATE	−0.69	0.14	18	1.36	.1909^AB^
	log_10_ *DA* × FSTE	0.79	0.07	292	−4.81	<.0001^b^
	log_10_ *DA* × PACO	0.94	0.08	292	−2.36	.0189^bc^
	log_10_ *DA* × PATE	1.00	0.08	292	−1.76	.08^ac^
$R_{m}^{2}$ = 0.67$R_{c}^{2}$ = 0.83						
(B) log_10_ *DH* ~ log_10_ *DA* + (species + Treat) random ID	Intercept (FSCO)	1.08	0.02	375	66.41	<.0001^A^
	log_10_ *DA* (slope) (FSCO)	0.20	0.01	375	24.24	<.0001
	FSTE	1.09	0.01	18	0.60	.5542^A^
	PACO	0.85	0.01	18	−17.23	<.0001^B^
	PATE	0.85	0.01	18	−17.18	<.0001^B^
$R_{m}^{2}$ = 0.87$R_{c}^{2}$ = 0.88						
(C) log_10_ *CWT* ~ log_10_ *DA* + Treat random ID	Intercept (PACO)	0.36	0.02	47	14.94	<.0001
	log_10_ *DA* (slope) (PACO)	0.04	0.01	47	3.03	.0039
	PATE	0.37	0.01	6	0.29	.7843
$R_{m}^{2}$ = 0.14$R_{c}^{2}$ = 0.24						
(D) log_10_ *CA*_*PHL*_ ~ log_10_ *DA* + (species + Treat) random ID	Intercept (FSCO)	1.32	0.13	99	10.45	<.0001^A^
	log_10_ *DA* (slope) (FSCO)	0.56	0.07	99	7.79	<.0001^a^
	FSTE	1.25	0.18	16	−0.37	.7147^A^
	PACO	1.53	0.19	16	1.06	.3034^A^
	PATE	1.53	0.18	16	1.15	.2664^A^
	log_10_ *DA* × FSTE	0.64	0.10	99	0.83	.411^a^
	log_10_ *DA* × PACO	0.17	0.11	99	−3.40	.001^b^
	log_10_ *DA* × PATE	0.14	0.11	99	−3.80	.0002^b^
$R_{m}^{2}$ = 0.79$R_{c}^{2}$ = 0.80						

The relationship of *Dh* with *DA* revealed a rigid axial scaling, significantly invariant across years and between treatments (CO and TE), with *F. sylvatica* having significantly larger conduits (i.e., higher *y*‐intercept) than *P. abies* (Figure 2; Table 1B).

**FIGURE 3:**
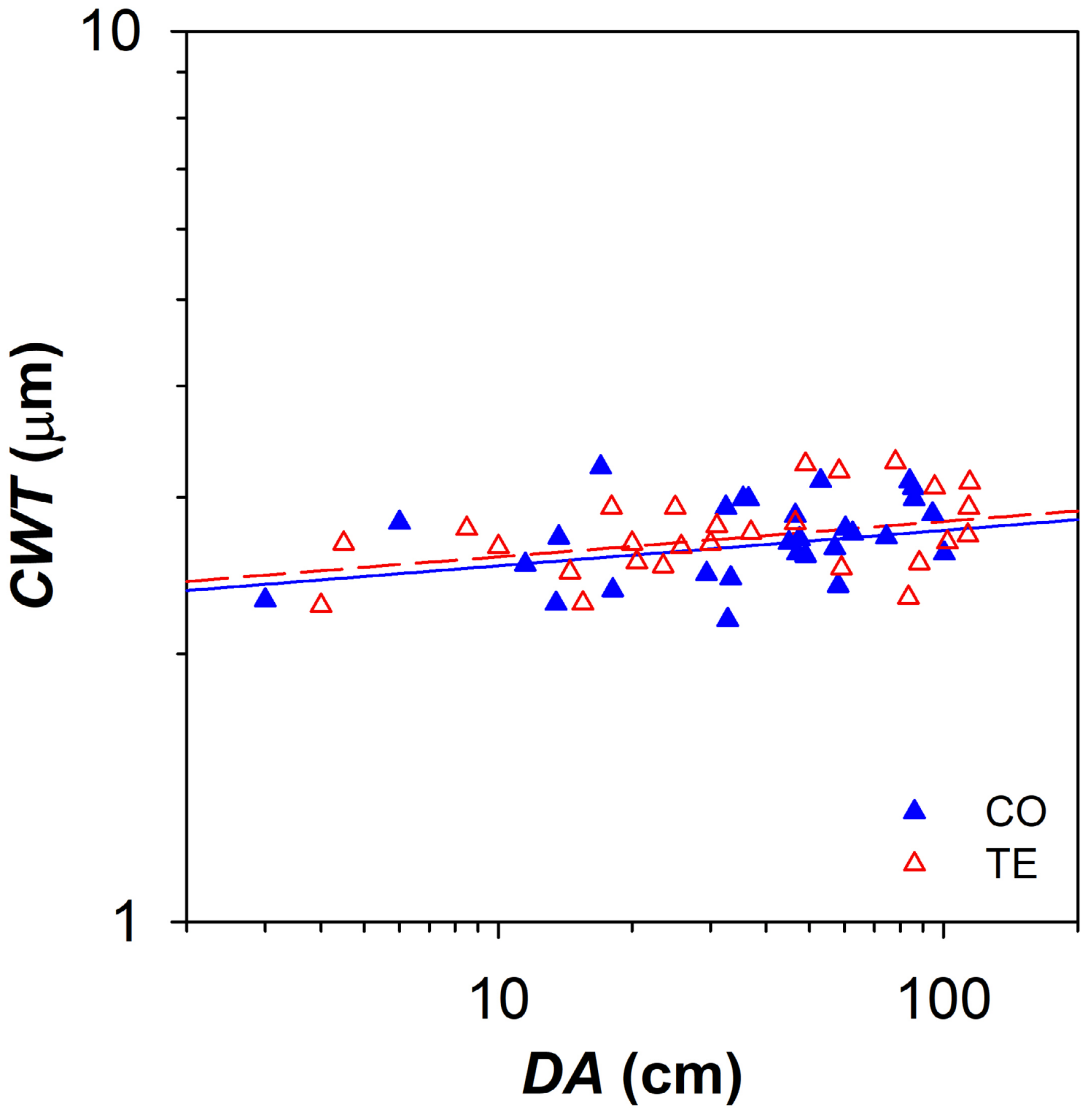
Variation in the thickness of tracheid cell walls (*CWT*) with the distance from the branch apex (*DA*) in control (CO: Filled blue triangles) and droughted (TE: Empty red triangles) for *P. abies*. Fitting lines (solid for CO and dashed for TE) are according to Table 1C.

The cell wall thickness (*CWT*) of *P. abies* tracheids slightly increased with *DA*, but showed no significant differences between CO and TE trees (Figure 3; Table 1C).

**FIGURE 4:**
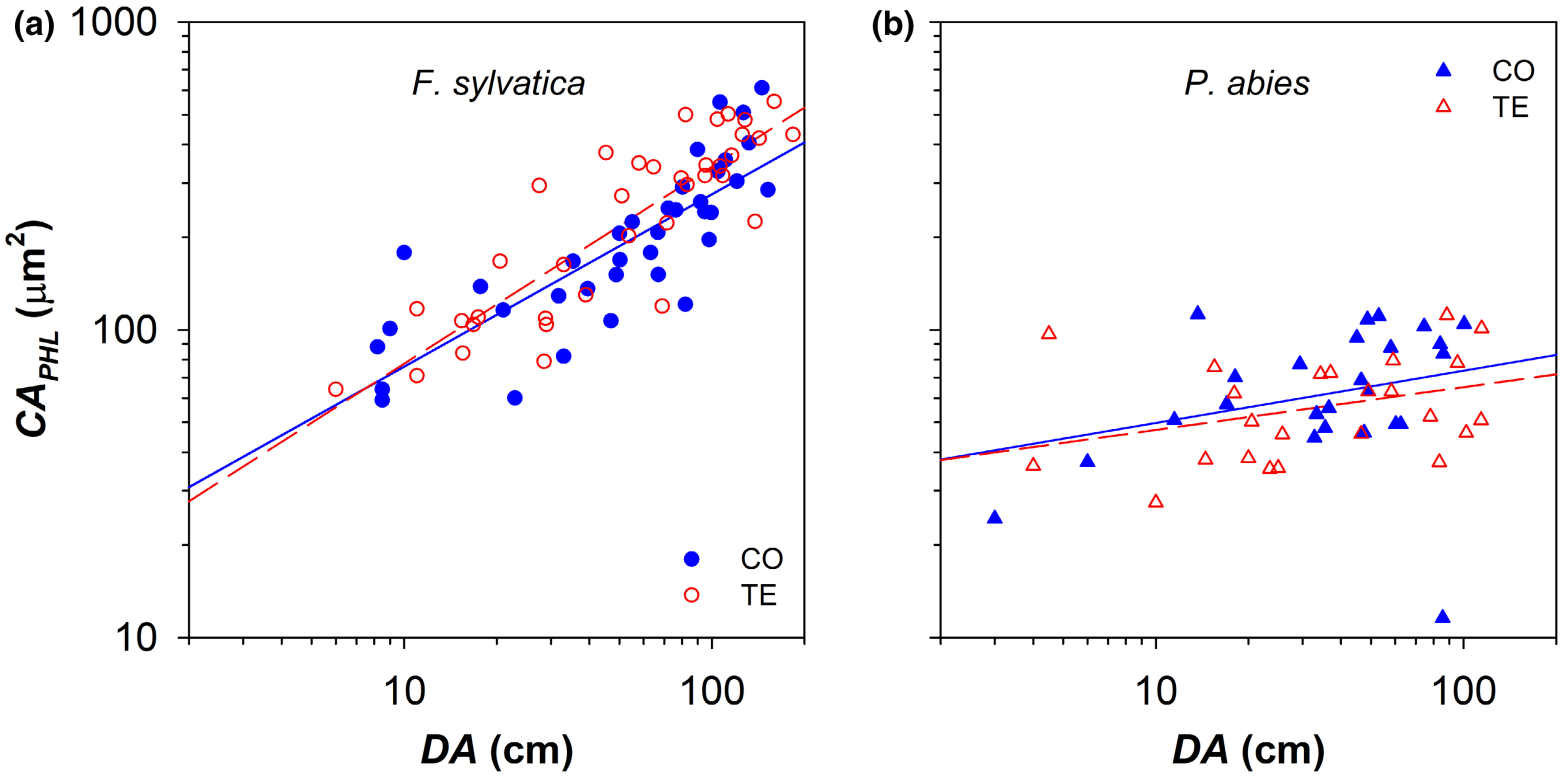
Variation in the mean lumen area of phloem sieve cells (*CA*_*PHL*_) with the distance from the branch apex (*DA*) in control (CO: Filled blue symbols) and droughted (TE: Empty red symbols) for (a) *F. sylvatica* (circles) and (b) *P. abies* (triangles). Fitting lines (solid for CO and dashed for TE) are according to Table 1D.

The axial variation of the lumen area of phloem sieve cells (*CA*_*PHL*_) with *DA* revealed species‐specific patterns with *y*‐intercept and slope significantly differing between *F. sylvatica* and *P. abies*, but no significant effects of precipitation exclusion in both scaling parameters (Figure 4; Table 1D).

### 3.2 Branch elongation

Since 2014, the annual branch elongation (*ΔL*) showed high intraspecific variability. Although an overall difference in *ΔL* between control and droughted trees for the whole period of precipitation exclusion in both species did not emerge, yet the lowest annual *ΔL* was observed in some TE trees in both species. Furthermore, in *F. sylvatica ΔL* was significantly lower in TE than CO trees in 2016, while in *P. abies* TE trees showed a significant trend of decreasing *ΔL* with time, although only in 2017 *ΔL* resulted significantly lower than control trees (Figure 7).

### 3.3 Allocation to leaf area and branch biomass

**FIGURE 5:**
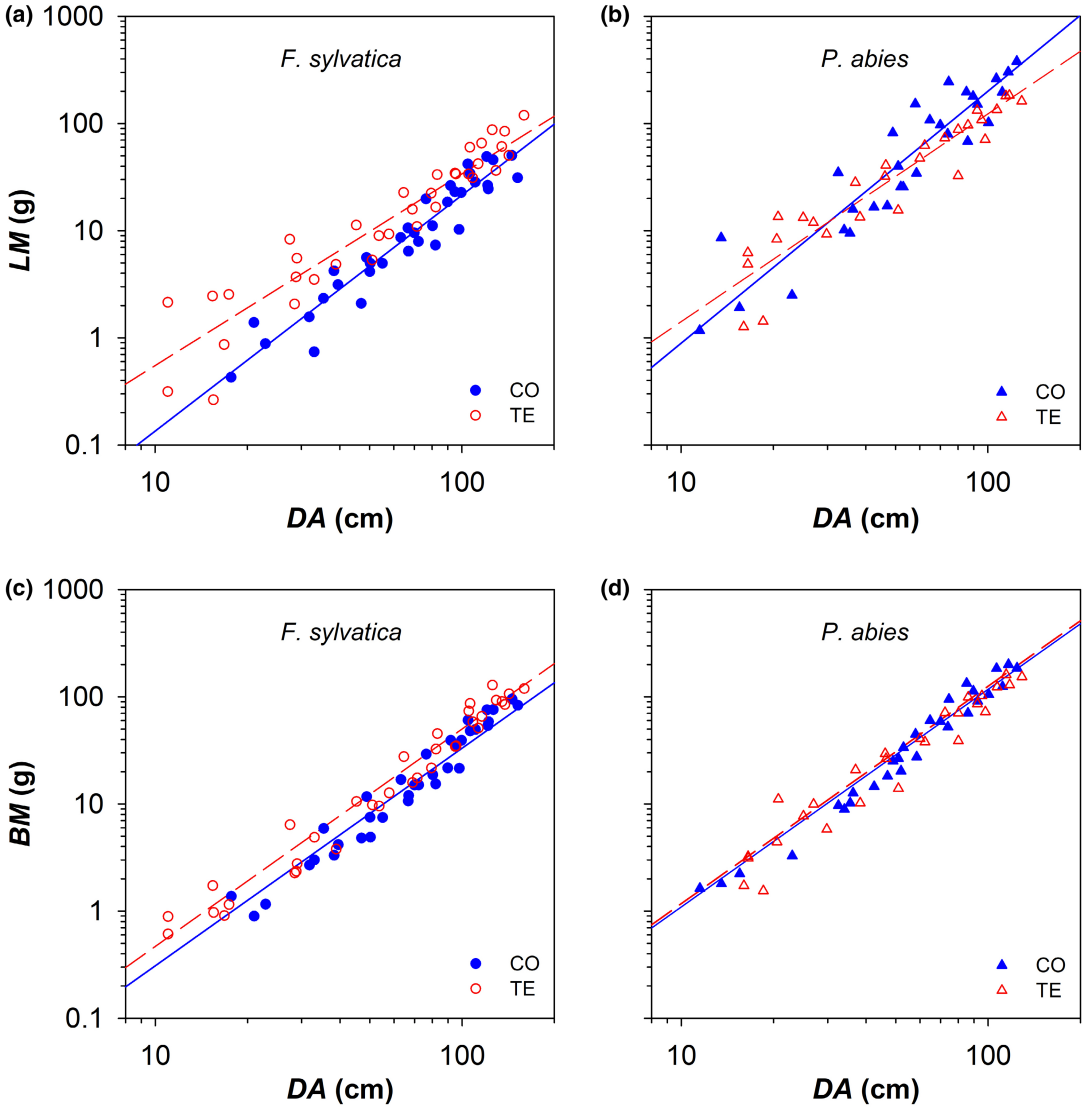
Basipetal patterns of allocation to leaf biomass (*LM*: a, b) and branch biomass (*BM*: c, d) along the main branch axis. Variation in *LM* cumulated along the branch axis starting from the apex with the distance from the branch apex (*DA*) (CO: Filled blue symbols) and droughted (TE: Empty red symbols) for (a, c) *F. sylvatica* (circles) and (b, d) *P. abies* (triangles). Fitting lines (solid for CO and dashed for TE) are according to Table 2A and C.

The total leaf mass that progressively accumulated starting from the branch apex along the main branch axis (*LM*) increased with *DA* according to a similar axial scaling between the CO trees of the two analysed species (significantly similar *y*‐intercept and slope). While throughfall precipitation exclusion did not affect the *LM* allocation pattern in *P. abies*, the most distal portion of the branch of *F. sylvatica* TE trees loaded more *LM* than CO trees did (corresponding to higher *y*‐intercept and lower slope) (Figure 5a and b; Table 2A).

**TABLE 2:** Results of the optimal linear mixed‐effect models predicting the effects of log_10_ *DA* and *species + treatment* on (A) log_10_ *LM*, (B) log_10_ *LA* and (C) log_10_ *BM*. (D) Results of the optimal linear mixed‐effect models predicting the effects of log_10_ *LA* and *species + treatment* on log_10_ *BM*. Tree ID was used as random factor in all models. Different letters indicate different estimates with significance at *p* < .05, or at *p* < .1 in case of symbol #. Abbreviations for species and treatments: FSCO Fagus Sylvatica COntrol, FSTE Fagus Sylvatica throughfall exclusion, PACO Picea Abies COntrol, PATE Picea Abies throughfall exclusion

Model	Covariates and fixed effects	Estimate	SE	*df*	*t*‐value	*p*‐value
(A) log_10_ *LM* ~ log_10_ *DA* + (species + Treat) random ID	Intercept (FSCO)	−3.07	0.20	102	−15.30	<.0001^A^
	Log_10_ *DA* (slope) (FSCO)	2.20	0.10	102	22.14	<.0001^a#^
	FSTE	−2.05	0.25	16	4.08	.0009^B^
	PACO	−2.40	0.28	16	2.41	.0282^A^
	PATE	−1.79	0.27	16	4.80	.0002^B^
	Log_10_ *DA* × FSTE	1.79	0.12	102	−3.34	.0012^b^
	Log_10_ *DA* × PACO	2.35	0.14	102	1.10	.2722^a^
	Log_10_ *DA* × PATE	1.94	0.13	102	−1.93	.0565^b#^
$R_{m}^{2}$ = 0.89$R_{c}^{2}$ = 0.96						
(B) log_10_ *LA* ~ log_10_ *DA* + (species + Treat) random ID	Intercept (FSCO)	−5.06	0.21	102	−24.05	<.0001^A^
	log_10_ *DA* (slope) (FSCO)	2.20	0.10	102	21.07	<.0001^a^
	FSTE	−3.88	0.26	16	4.49	.0004^B^
	PACO	−4.76	0.29	16	1.01	.3295^A^
	PATE	−4.2	0.28	16	3.07	.0074^B^
	log_10_ *DA* × FSTE	1.75	0.13	102	−3.55	.0006^b^
	log_10_ *DA* × PACO	2.29	0.15	102	0.59	.5567^a#^
	log_10_ *DA* × PATE	1.88	0.14	102	−2.26	.0259^b#^
$R_{m}^{2}$ = 0.85$R_{c}^{2}$ = 0.95						
(C) log_10_ *BM* ~ log_10_ *DA* + (species + Treat) random ID	Intercept (FSCO)	−2.54	0.08	99	−32.01	<.0001^A^
	log_10_ *DA* (slope) (FSCO)	2.03	0.04	99	54.86	<.0001
	FSTE	−2.36	0.06	18	3.06	.0067^B^
	PACO	−1.99	0.06	18	8.94	<.0001^C^
	PATE	−1.96	0.06	18	9.15	<.0001^C^
$R_{m}^{2}$ = 0.95$R_{c}^{2}$ = 0.98						
(D) log_10_ *BM* ~ log_10_ *LA* + (species + Treat) random ID	Intercept (FSCO)	2.24	0.07	99	32.13	<.0001^A#^
	log_10_ *LA* (slope) (FSCO)	1.01	0.02	99	45.43	<.0001
	FSTE	1.97	0.09	18	−2.89	.0097^B^
	PACO	2.31	0.10	18	0.77	.4522^C^
	PATE	2.42	0.10	18	1.87	.0776^C#^
$R_{m}^{2}$ = 0.90$R_{c}^{2}$ = 0.95						

The total branch biomass that progressively accumulated starting from the branch apex along the main branch axis (*BM*) scaled to the second power (*b* = 2) of *DA* irrespective of species and treatment (Figure 5b). Species differed for a higher *BM* in *P. abies* (higher *y*‐intercept). A treatment effect was observed only in *F. sylvatica*, with TE trees loading more *BM* along the branch axis (i.e., higher *y*‐intercept) than CO trees (Figure 5c and d; Table 2C).

**FIGURE 6:**
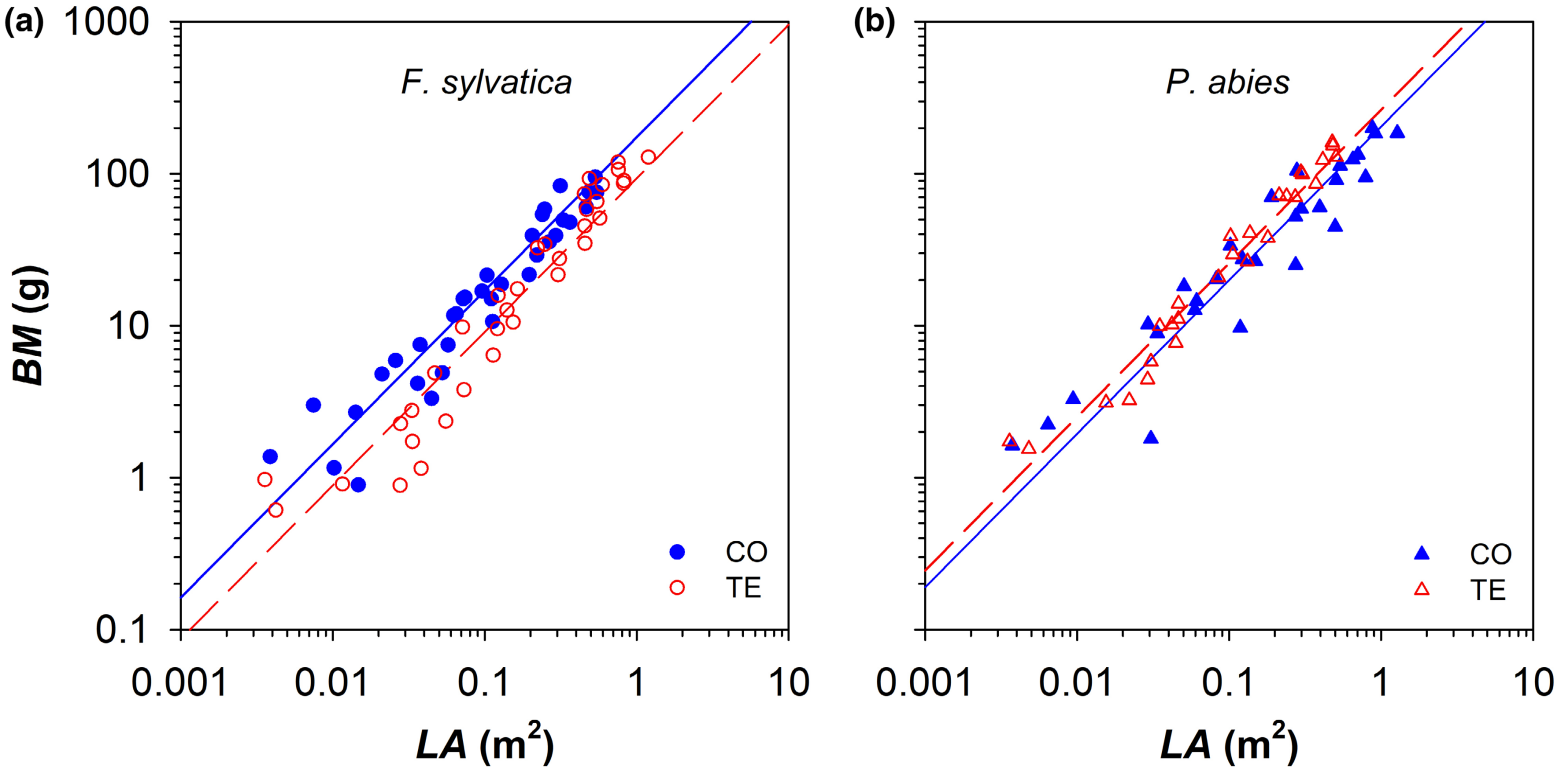
Relationship between the total branch biomass cumulated starting from the branch apex (*BM*) and the total leaf area cumulated starting from the branch apex (*LA*) in control (CO: Filled blue symbols) and droughted (TE: Empty red symbols) for (a) *F. sylvatica* (circles) and (b) *P. abies* (triangles). Fitting lines (solid for CO and dashed for TE) are according to Table 2D.

The allometric relationship expressing the biomass partitioning between leaf area and branch biomass (i.e., *BM* vs. *LA*) was significantly similar (i.e., same *y*‐intercept and slope) between the CO trees of both species. While precipitation exclusion did not affect the allocation pattern in *P. abies*, *F. sylvatica* TE trees showed a significantly higher *y*‐intercept and lower slope, corresponding to distal branches with a lower ratio *BM*:*LA* (Figure 6; Table 2D).

**FIGURE 7:**
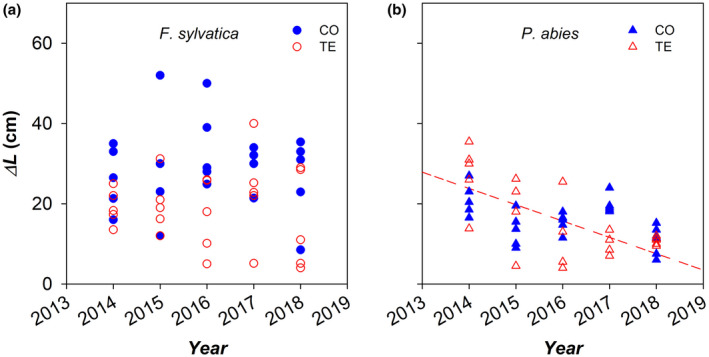
Annual variation of the axial elongation (*ΔL*) in the analysed top branches of control (CO: Filled blue symbols) and droughted (TE: Empty red symbols) (a) *F. sylvatica* (circles) and (b) *P. abies* (triangles) trees during the whole experimental period (2014–2018) of throughfall precipitation exclusion.

**FIGURE 8:**
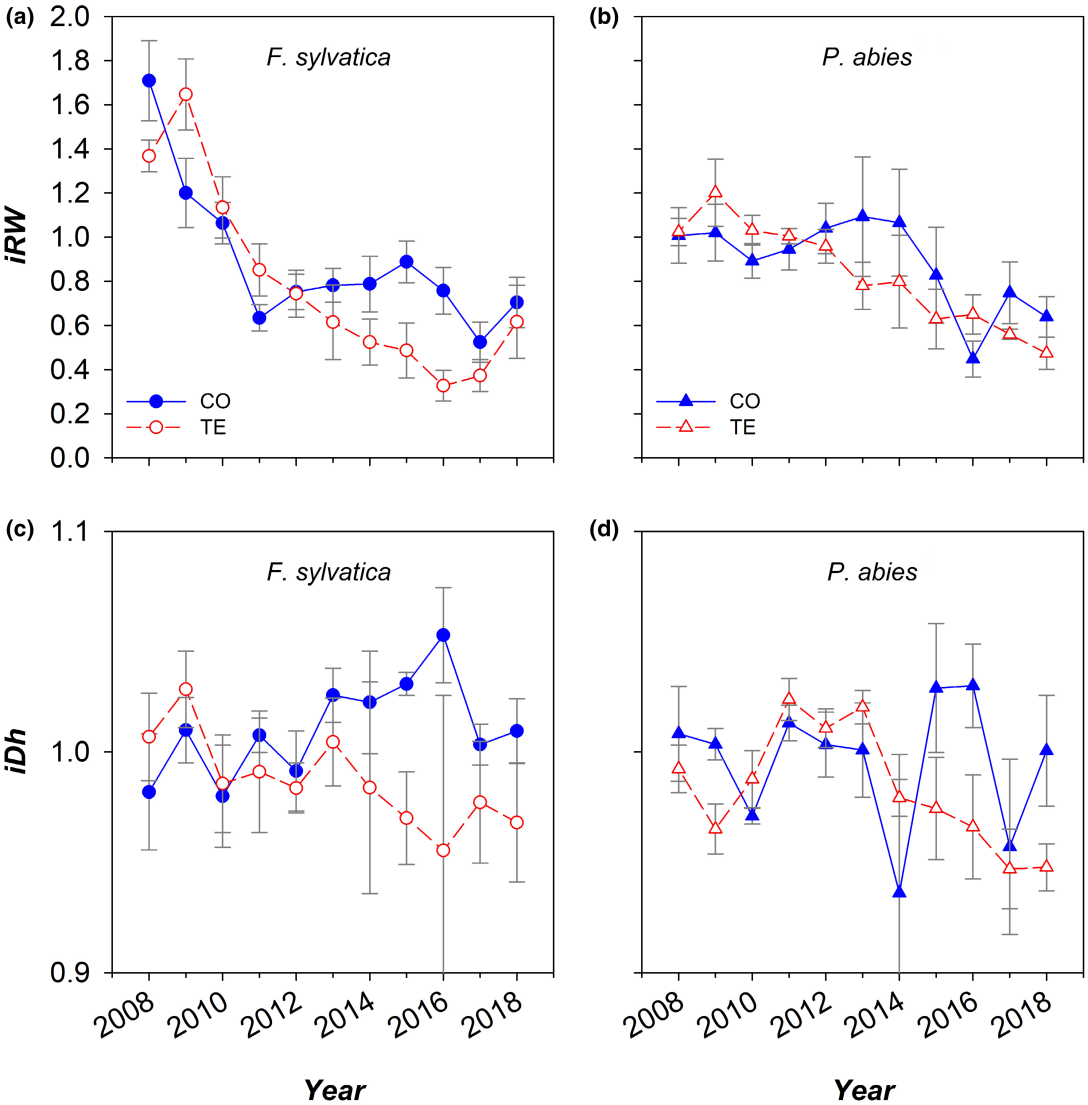
Mean annual variation of the indexed ring width (*iRW*: a, b) and the indexed hydraulically weighted diameter of xylem conduits (*iDH*) in the analysed stem cores of control (CO: Filled blue symbols) and droughted (TE: Empty red symbols) (a, c) *F. sylvatica* (circles) and (b, d) *P. abies* (triangles) trees. *iRW* and *iDh* are relative values calculated over the reference period 2008–2013 (i.e., the 5 years preceding the drought experiment). Error bars indicate standard errors.

### 3.4 Stem diameter growth and xylem anatomy

The 10 outermost annual xylem rings of the stem showed a progressive reduction in mean width (*RW*) with time in both species and treatments. However, TE trees appeared to diverge from CO trees starting from the beginning of the throughfall exclusion in 2014, showing a progressive and sharper decline in *RW* (Figure 8a and b). At the anatomy level, the hydraulically weighted diameter of xylem conduits (*Dh*) did not vary significantly across years, although a slight, but not significant, decline since 2014 was emergent in the TE trees of both species (Figure 8c and d).

## 4 DISCUSSION

In this study, we carried out different analyses aimed at understanding the possible patterns of morphological/anatomical acclimation to decreased water availability in coexisting *F. sylvatica* and *P. abies* mature trees. We investigated whether trees exposed to 5 years of throughfall precipitation exclusion during the growing season (i) modified the conductive architecture of xylem and phloem, (ii) reduced/increased the C investment into new biomass of leaves and branch/stem tissues, and (iii) modified the C costs associated to the maintenance respiration of living tissues.

### 4.1 Conservativism of xylem anatomy at the branch level

*Dh* increased with the distance from the branch apex according to a power scaling with exponent *b* = 0.2 independent of species and treatment. Such a result is consistent with the hypothesis of strong conservativism of the axial xylem design during ontogeny (Prendin, Petit, et al., 2018) and across species and environmental conditions (Anfodillo et al., 2013; Olson et al., 2014). According to Hagen–Poiseuille, the conductance of a given xylem conduit scales with the fourth power of its diameter (Tyree et al., 1991), and the resistance to drought‐induced cavitation has been commonly reported to decrease with increasing conduit diameters (Hacke et al., 2006; Larter et al., 2017; Nardini et al., 2017; Pittermann et al., 2006). The typical xylem's axial configuration has been proposed to represent the optimal gain‐cost solution for maximizing the hydraulic conductance and the resistance to drought‐induced embolism formation for a given C investment for the build‐up of the long distance transport network (Mencuccini et al., 2007). Such a hypothesis would imply that the production of a more embolism resistant xylem while contextually maintaining the total xylem conductance would increase the C costs associated to the tissue production because more conductive cells should be formed to compensate for the lower conductance of their narrower lumen diameter. At the other extreme, larger and more efficient conduits would allow a lower C investment into the xylem tissue to maintain the total xylem conductance, at the cost of likely increasing the vulnerability to air embolism.

Net of the axial conduit widening, *Dh* resulted larger in the *Fagus sylvatica* vessels compared with the spruce tracheids (i.e., higher *y*‐intercept in the allometric relationship *log*_*10*_ *Dh = a + b × log*_*10*_ *DA*), but it was not affected by the artificial drought in the analysed branches of both species. This result is consistent with other anatomical studies reporting the axial variation of xylem conduit diameter along stem or branches. These studies showed that trees growing under low water availability produce either marginally (Guérin et al., 2018; Kiorapostolou et al., 2018; Kiorapostolou et al., 2020, Kiorapostolou & Petit, 2019) or substantially (Petit et al., 2016) larger xylem conduits compared with those in moister conditions. In particular, results from a long‐term experiment of 50% exclusion of incoming precipitation in a dry mountain piñon‐juniper dry forest revealed a marginal increase in the tracheid lumen area in the analysed *Pinus edulis* (Engelm.) in response to the chronic reduction in water availability (Guérin et al., 2018). Contextually, it was found that an episodic drought occurring during the experiment at the time of intense cambial activity negatively affected the final conduit diameter (Guérin et al., 2018). Similar results were obtained by Kiorapostolou et al. (2020), reporting slightly larger tracheids in declining mature trees in a Scots pine stand in the Iberian peninsula.

On the contrary, a large body of published studies reported analyses commonly carried out applying a punctual sampling approach, i.e., measurements taken at one single position along the branch/stem axis, selected at the organ (branch/stem) base, or on the basis of the branch age (i.e., number of xylem rings: typically 2–3) or diameter (typically 1–2 cm). This type of sampling procedure is pervasive in ecological studies, but substantially neglects the (possible) existence of axial variations in several xylem anatomical traits. Consequently, the wide body of data obtained with a punctual sampling approach as described above can likely and tragically represent a large source of flawed results. No longer it should be neglected that trees sampled according to the classical punctual approach most likely would show narrower and more embolism resistant conduits in case of short stature and if the axial stem/branch elongation is slow (like trees under dry conditions): samples would be simply taken at a shorter distance from the stem/branch apex compared with those in taller and/or faster growing trees.

The thickness of the tracheid cell walls (*CWT*) has been proposed to contribute, in tandem with the conduit's lumen diameter (*d*), to the mechanical resistance against cell wall collapse under the effect of xylem tension ([2 × *CWT*/*d*]^2^: Hacke et al., 2001). In the analysed *P. abies* branches, we found no evidence that *CWT* was affected by artificial drought. It was characterized by a significant and rather flat axial trend (*b* = 0.05), although the statistical model revealed that the distance from the branch apex did not explain the majority of the trait variance ($R_{c}^{2}$ = 0.24), consistent with a previous report (Prendin, Petit, et al., 2018).

Since in the analysed branches neither *CWT* nor *Dh* were significantly affected by the applied precipitation exclusion in both species and that *Dh* varied axially at a faster rate than *CWT*, this would suggest that variations in implosion resistance mostly depends on the *Dh* patterns. Again, all else being equal, analyses based on classical punctual sampling most likely would reveal higher resistance against implosion in short and slow growing trees, as commonly reported for trees growing under water limiting conditions (Barigah et al., 2013; Rowland et al., 2015).

### 4.2 Conservativism of phloem anatomy at the branch level

Phloem sieve elements increased in lumen area (*CA*_*PHL*_) axially with increasing *DA*. The axial scaling slightly differed between *F. sylvatica* and *P. abies*, but in the range of those reported in literature. Altogether, the available data would suggest a convergent axial scaling of *CA*_*PHL*_ across species, characterized by an exponent in the range of that reported for the xylem conduit diameter (*b ~* 0.1–0.3) (Jyske & Hölttä, 2015; Kiorapostolou et al., 2020; Kiorapostolou & Petit, 2019; Petit & Crivellaro, 2014; Savage et al., 2017). In fact, according to the Munch's circulation hypothesis, the transport of phloem sap should be coordinated to that of water along the xylem (Hölttä et al., 2006).

In the analysed branches of *F. sylvatica* and *P. abies*, *CA*_*PHL*_ was not affected by artificial drought, suggesting no acclimation of phloem anatomy to drought. Phloem sap conductance can be negatively affected by a drought‐related increase in sap viscosity due to tissue osmoregulation or/and dehydration (Sevanto, 2014). Consistently, the phloem sap velocity has been reported to decrease by nearly 50% in the droughted compared with control *Fagus sylvatica* trees (Hesse et al., 2019), although NSC concentration was reported not to differ between treatments (Hesse et al., 2021). However, we did not measure the area of conductive phloem, and therefore no definitive conclusion can be made on wheater a larger phloem tissue area was produced under drought to compensate for the potential limitations to phloem transport due to the likely higher sap viscosity. In a few studies accounting for the path length effects on phloem anatomy, the phloem area and the lumen area of sieve elements resulted to be larger at drier sites (Kiorapostolou & Petit, 2019), and in trees showing signs of drought‐ induced decline of vigour (Kiorapostolou et al., 2020). Thus, in a carbon‐starvation scenario, where the NSC may decrease drastically (Hesse et al., 2021) the ability to transport and mobilize carbon in different tissues, plays a key role for the survival of the trees at the cost of reduced growth. On the contrary, small beech trees exposed to a long‐term drought treatment have been reported to produce narrower sieve tubes at the stem base (i.e., by applying the classical punctual sampling approach) (Dannoura et al., 2019).

### 4.3 Allocation patterns of leaf and xylem biomass at the branch level

Leaf biomass provides with photosynthesis, the necessary resources to maintain a long‐term positive C balance, when stored C resources can sustain physiological needs in case of lack of resources. Besides, its production and maintenance represent relevant energetic costs. Therefore, it has been proposed that plants acclimate to conditions of limiting stomatal conductance by increasing the total leaf area while reducing the allocation to current axial and radial growth (Anfodillo et al., 2016). Indeed, recent empirical measurements seemed to support this hypothesis (Anfodillo et al., 2016; Kiorapostolou et al., 2018; Kiorapostolou & Petit, 2019; Petit et al., 2016).

The analysed *F. sylvatica* and *P. abies* trees differed in axial and radial growth. The branch elongation rate was higher in beech (~34.5 cm/year) compared with spruce (~23.5 cm/year) with an overall trend of branch elongation reduction, but the high intra‐specific variability did not present any statistically significant effect made by prolonged drought. On the contrary, the radial increment of both top branches and stem base was higher in *P. abies* than *F. sylvatica*, and it was differentially affected in the two species.

The total loading of leaf/needle biomass (*LM*) along the analysed top branches was similar in control beech and spruce trees. *LM* cumulated progressively with increasing *DA* according to the same power scaling (i.e., exponent *b* of ~2.2–2.3). Both species responded to precipitation exclusion by increasing the allocation to *LM* within the first ~1 m from the apex of the main branch axis (Table 2). Furthermore, leaves from stressed *F. sylvatica* trees were characterized by a significantly lower leaf mass per area (*LMA* = leaf dry weight/area of leaf lamina), which amplified the differences between treatments in the allocation to light interception (i.e., in leaf area, *LA*: Table 2). Notably, such a plastic response did not characterize the droughted spruce trees, and it is not in agreement with most literature data, reporting increased *LMA* with reducing soil water availability (Poorter et al., 2009). However, the *LMA* response in the droughted beech trees was consistent with another study reporting decreased *LMA* of canopy leaves in response to prolonged experimental drought (Kuang et al., 2017).

Along the main axis of the analysed top branches, the ring area (*RA*) of the outermost five rings (2014–2018) was significantly higher within the first ~1 m from the apex in the droughted compared with the control beech trees. This resulted in an overall higher branch biomass (*BM*) within the ~1 m from the branch apex. Instead, *RA* and *BM* in spruce top branches were not affected by artificial drought. Since the structural changes in *F. sylvatica* did not result in higher hydraulic, it could be speculated the larger branch biomass in the distal branch portion of droughted beech trees likely provided the mechanical requirements for sustaining the larger and heavier leaf area.

These results would suggest that droughted beech trees increased the leaf area per unit of branch axis length to maintain the total leaf area, and contextually reduced the C cost associated to the total biomass production by decreasing *LMA*, and the radial growth towards the stem base.

The width of xylem rings at breast height showed an overall progressive decline in both *F. sylvatica* and *P. abies*. However, no significant differences emerged between treatments, except for the narrower vessels of 2015 and the narrower rings of 2016 in *F. sylvatica*.

The progressive decline of xylem ring width and mean conduit lumen area at the stem base have been reported to characterize the growth of trees eventually succumbing even decades after the predisposing drought events (Cailleret et al., 2017; Pellizzari et al., 2016). Notably, the reduction in the xylem conductivity at the stem base unlikely would cause strong limitations to water transport, since the contribution of these tissues to the total hydraulic resistance is negligible compared with those towards the crown periphery (Lechthaler et al., 2020; Prendin, Mayr, et al., 2018).

### 4.4 Maintenance costs of branch biomass

The relationship of *BM* vs. *LA* can describe the maintenance cost of the branch living biomass for a given leaf area. Indeed, the assessed scaling relationship was isometric (exponent *b* = 1): i.e., *BM* and *LA* vary with the same proportions.

The branch biomass associated with a given leaf area in the analysed top branches was higher in *P. abies* than *F. sylvatica*. All else being equal, this would suggest that the maintenance respiration cost of the living branch biomass is relatively more expensive in *P. abies* than *F. sylvatica*.

To summarize, while the allocation patterns to *LA* and *BM* were not affected by treatments, droughted beech trees allocated relatively more C to the production of new and more expanded leaves compared with the allocation to the supporting branch biomass. Results would then suggest that beech trees reacted to drought by reducing the C contribution that a unit leaf must provide to sustain the maintenance respiration of the total living body mass. For spruce, allocation patterns of *LA* and *BM* along the analysed branches were not significantly affected by the precipitation exclusion. Furthermore, these branches did not reveal any acclimation strategy in the their topmost part, but supported a more expensive maintenance of the living branch biomass.

In conclusion, although drought‐induced xylem embolism has been clearly demonstrated to play a key factor in leading a plant to death (Barigah et al., 2013; Rowland et al., 2015), yet our study contributed to increase the body of empirical evidence not supporting the hypothesis that acclimation to drought can be achieved by means of the production of a more embolism resistant xylem. On the contrary, the outcomes of this experiment of long‐term throughfall precipitation exclusion suggested that drought more negatively affected the C balance of *P. abies* than *F. sylvatica* trees. Beech trees compensated for the negative effects of reduced soil water availability on stomatal conductance and gas exchanges by lowering the minimum leaf water potential (Tomasella et al., 2018), and decreased the C cost associated to the production and maintenance of the branch biomass. Instead, *P. abies* substantially showed no signs of acclimation to drought, possibly exposing the species to a greater risk of mortality, that actually occurred (Pretzsch et al., 2020).

## CONFLICT OF INTEREST

None.

## AUTHOR CONTRIBUTIONS

GP and KHH designed the experiment. GP, KHH and BDH carried out the field sampling. KHH and BDH performed the measurements of tissues‘biomass. GP and DZ performed the anatomical measurements, all statistical analyses. GP and DZ wrote the manuscript draft, and all coauthors actively contributed to the final version.

## Data Availability

The data that support the findings of this study are available at Research Data UNIPD (https://doi.org/10.25430/researchdata.cab.unipd.it.00000639).
